# Genetic Structure of Populations of *Rhizoctonia solani* Anastomosis Group (AG)-2-2IIIB and AG-4HGI Causing Sugar Beet Root Diseases in China

**DOI:** 10.3390/jof12020097

**Published:** 2026-01-30

**Authors:** Can Zhao, Zhiqing Yan, Pengfei Li, Chenggui Han, Anpei Yang, Xuehong Wu

**Affiliations:** 1 College of Grassland Science and Technology, China Agricultural University, Haidian District, Beijing 100193, China; zhaocan2023@cau.edu.cn (C.Z.); xiaobaiyan1005@163.com (Z.Y.); 2 College of Plant Protection, China Agricultural University, Haidian District, Beijing 100193, China; 15248024114@163.com (P.L.); hanchenggui@cau.edu.cn (C.H.); 3 Institute of Plant Protection, Xinjiang Academy of Agricultural Science, Urumqi 830091, China; yap2002@126.com

**Keywords:** *Rhizoctonia solani*, AG-2-2IIIB, AG-4HGI, sugar beet, population genetic structure, simple sequence repeat

## Abstract

*Rhizoctonia solani* anastomosis group (AG)-2-2IIIB and AG-4HGI are the main pathogens causing sugar beet seedling damping-off and crown and root rot disease. In this study, 1232 loci of simple sequence repeats (SSRs) were obtained via transcriptome sequencing, with 592 from AG-2-2IIIB and 640 from AG-4HGI. Fourteen and twenty loci of SSRs were selected for studying the genetic structure of the AG-2-2IIIB and AG-4HGI populations, respectively. A population of 134 strains of AG-2-2IIIB and 145 strains of AG-4HGI, sampled from three geographic regions in China, indicated that both AG-2-2IIIB and AG-4HGI had a high level of genetic diversity, and that the selected SSR markers could reliably capture the genetic variation. Genetic analysis indicated that the individual strains of AG-2-2IIIB and AG-4HGI randomly mated within their respective population, and that a considerable degree of inbreeding was present among the populations. High to moderate gene flow and low to moderate population subdivision were detected among the populations of AG-2-2IIIB and AG-4HGI, which indicated that weak differentiation existed in these two subgroups. In addition, a founder effect (genetic drift) or a bottleneck effect was inferred to have occurred in the AG-4HGI population. This study provides the first analysis of the population genetic structure of AG-2-2IIIB and AG-4HGI associated with sugar beet seedling damping-off and crown and root rot disease, and the present results offer useful guidance for developing effective integrated disease management.

## 1. Introduction

Sugar beet (*Beta vulgaris* L.) is one of the world’s major sugar-yielding crops and second to sugarcane in global sugar production, which is mainly grown in temperate regions [1,2,3,4]. Despite its economic importance, sugar beet is frequently threatened by several pathogens that cause a series of diseases. Among them, seedling damping-off and crown and root rot caused by *Rhizoctonia* are destructive fungal diseases, posing serious threats to sugar beet growers in all sugar beet-growing regions worldwide, leading to both yield and quality losses [5,6,7,8,9]. If no effective control strategies are taken, these two diseases alone can cause a yield loss of 50% or more [10].

*Rhizoctonia solani*, the main pathogen associated with sugar beet seedling damping-off and crown and root rot, is a species complex composed of different anastomosis groups (AGs) [11]. At present, fourteen AGs (from AG-1 to AG-13 and AG-BI) have been identified, several of which are further divided into subgroups based on colony morphology, pathogenicity, host range, and biochemical and genetic properties [12,13,14,15]. Among these AGs or subgroups, AG-2-2IIIB and AG-4 (HGI, HGII, and HGIII) are major causal agents that threaten sugar beet growth worldwide, with AG-2-2IIIB mainly responsible for crown and root rot while AG-4 causes both damping-off and crown and root rot [15,16,17,18,19].

Because *R. solani* has a major impact on sugar beet yield and quality, a range of strategies have been implemented for disease management, such as cultural practices, fungicide applications to seed, soil, or sugar beet plants, and the use of resistant cultivars. However, the method of rotating sugar beet crops with others provides incomplete control, as AG-2-2IIIB and AG-4 have broad host ranges and can survive in the soil as mycelia and sclerotia for a long time [6,13,14,20]. Chemical control is an efficient strategy used in some sugar beet-cultivating regions, but the number of commercially available chemicals remains limited [21,22]. Azoxystrobin was first registered for use on sugar beet in 1999, and showed excellent efficiency against *Rhizoctonia* disease of sugar beet [23,24,25]. However, this quinone-outside inhibitor fungicide has a high risk of resistance development due to the site-specific mode of action, and several cases of *Rhizoctonia* resistant to this fungicide have already been reported [26,27,28]. Although considerable progress has been made in improving sugar beet resistance to *R. solani*, no immune sugar beet germplasms have yet been found [29]. Significant efforts have been made to search for *R. solani*-resistant beet germplasms, but mapping data generated by breeders indicate that the resistant loci are somehow associated with undesirable traits, such as reduced yield [22].

Understanding the population genetic structure of plant pathogens provides insight into their life history, evolutionary potential, modes of reproduction, and global migrations within agroecosystems for adaptation, which is useful for developing effective integrated disease management strategies [30]. Due to the different environmental conditions and stresses, such as geographic isolation, crop cultivation pattern alteration, and fungicide use, the genetic diversity of *R. solani* is highly differentiated. Therefore, insights into the population dynamics and the epidemiology of *R. solani* are beneficial for the control of sugar beet seedling damping-off and crown and root rot disease.

The introduction and application of molecular markers has advanced the species classification of plant pathogens and improved our understanding of their population biology. To date, many molecular marker techniques have been developed by researchers for fingerprinting and genetic analysis in the field of plant pathology, such as inter simple sequence repeats (ISSRs) [31,32], simple sequence repeats (SSRs) [33,34,35], restriction fragment length polymorphism (RFLP) [36], random amplified polymorphic DNA (RAPD) [37], and amplified fragment length polymorphism (AFLP) [38]. Among these techniques, SSRs-also known as microsatellite sequences (MSs) or short tandem repeats (STRs)-are widely used to analyze the population genetic diversity of phytopathogens because of their high resolution and high levels of polymorphism.

A high level of genetic variation has been reported in *R. solani* AG-1 IA, AG-3, and *R. cerealis* AG-DI, suggesting that a cryptic sexual stage dominates these AGs of *Rhizoctonia* [33,34,39]. High levels of genetic variation and evidence of sexual reproduction have also been reported in AG-2-2IIIB isolated from soybean in North America [14] and creeping bentgrass in Canada [40], as well as AG-4HGI isolated from diseased roots, crowns, and hypocotyls of cucumber, aubergine, pepper, snap bean, squash, and sugar beet in Iran [41]. However, information on the population structure of AG-2-2IIIB and AG-4HGI causing sugar beet root disease in China remains lacking. Thus, in this study, we aimed to develop a repertoire of primer pairs that could amplify polymorphic microsatellite loci in *R. solani* AG-2-2IIIB and AG-4HGI, and to employ these SSR primers to analyze the population genetics of AG-2-2IIIB and AG-4HGI associated with sugar beet seedling damping-off as well as crown and root rot disease in China.

## 2. Materials and Methods

### 2.1. Fungal Strains and Populations

**Figure 1:**
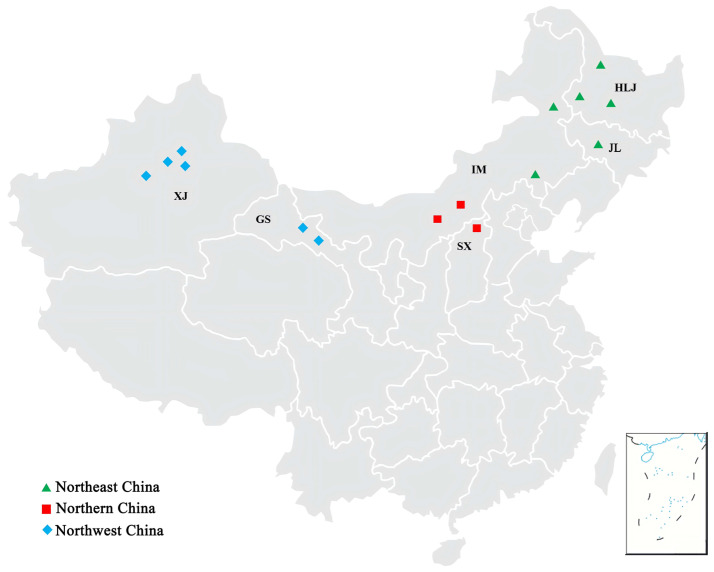
Geographic locations of the three sugar beet-growing regions where *Rhizocronia solani* AG-2-2IIIB and AG-4HGI strains were collected for use in this study. Abbreviation and full name of provinces and autonomous regions are as follows: HLJ, Heilongjiang province; JL, Jilin province; IM, Inner Mongolia autonomous region; SX, Shanxi province; GS, Gansu province; XJ, Xinjiang Uygur autonomous region.

Diseased sugar beet seedlings and roots exhibiting typical symptoms of either damping-off or crown and root rot were collected from 15 sampling locations across the main cultivation regions in China: Heilongjiang province, Jilin province, Shanxi province, Gansu province, Inner Mongolia autonomous region, and Xinjiang Uygur autonomous region (Figure 1). *Rhizoctonia* isolates were isolated from the diseased samples and then identified based on the morphological characteristics and sequence analysis of the internal transcribed spacers of the ribosomal DNA (rDNA ITS) [15,19]. In total, 134 AG-2-2IIIB (Table S1) and 145 AG-4HGI (Table S2) strains of *R. solani* were collected and identified, and subsequently used for population genetic structure analysis in this study. Based on geography, climate, and agricultural practices, the sampling locations from the six provinces or autonomous regions were grouped into three sugar beet-growing regions in China, i.e., Northeast China (NE) comprising Heilongjiang, Jilin province, and Chifeng city and Hinggan League of Inner Mongolia autonomous region; Northern China (NC), comprising Shanxi province, Ulanqab city and Baotou city of Inner Mongolia autonomous region; and Northwest China (NW), comprising Gansu province and Xinjiang Uygur autonomous region (Figure 1). The geographic regions are separated from each other by distances exceeding 500 km. All the tested 134 AG-2-2IIIB and 145 AG-4HGI strains were grown on potato dextrose agar plates covered with cellophane film membranes (PDA-CF) for 5 d, and then the mycelia were collected for genomic DNA extraction.

### 2.2. cDNA Library Preparation and Transcriptome Analysis

The AG-2-2IIIB strain RN158 and AG-4HGI strain HLJ-22 were randomly selected from the 134 identified AG-2-2IIIB strains and 145 identified AG-4HGI strains, respectively, and subsequently cultured on PDA-CF plates for 5 d in the dark with three replicates. Then, 0.2 g of mycelia was collected from each replication for RNA extraction using TRIpure Reagent (Aidlab Biotechnologies, Beijing, China) according to the manufacturer’s instructions. RNA concentration and purity were measured using NanoDrop 2000 (Thermo Fisher Scientific, Wilmington, DE, USA). RNA integrity was assessed using the RNA Nano 6000 Assay Kit of the Agilent Bioanalyzer 2100 system (Agilent Technologies, Santa Clara, CA, USA). The cDNA library of pooled RNA was constructed via the method described by Yang et al. [42]. The resulting AG-2-2IIIB and AG-4HGI cDNA library was sequenced using an Illumina HiSeq 4000 sequencing platform at Beijing Biomarker Technologies Co., Ltd. (Beijing, China).

Clean data (clean reads) were obtained by removing reads containing adapters and ploy-N sequences, as well as low-quality reads from raw data. Subsequently, clean reads were assembled using Trinity [43]. To annotate the AG-2-2IIIB and AG-4HGI transcriptome, unigenes were searched against the following databases: Nr protein (NCBI non-redundant protein sequences), Swiss-Prot (a manually annotated and reviewed protein sequence database) [44], KOG (euKaryotic Orthologous Groups) [45], KEGG (Kyoto Encyclopedia of Genes and Genomes) [46], and Pfam (Protein family) [47].

### 2.3. SSR Loci Development and Primer Design

SSR loci were identified from the AG-2-2IIIB and AG-4HGI transcriptome sequence data using MISA (MIcroSAtellite identification tool) and SAMtools, and the search criterion was defined as follows: a minimum number of repeats of ten for mono-nucleotide repeats, six for dinucleotide repeats, five for trinucleotide repeats, and three for tetra-, penta-, and hexa-nucleotide repeats [48]. Subsequently, SSR primers were designed based on the SSR flanking sequences using Primer Premier 5.0 software (PREMIER Biosoft International, Palo Alto, CA, USA) according to the following criteria: primer length of 16–22 bp, PCR product size of 100–300 bp, annealing temperature of 40–60 °C, and GC content of 40–60% [42]. The primer pairs designed for SSR markers were used to screen for successful amplification using polymerase chain reaction (PCR) and for fragment length polymorphisms using a subset of the tested strains. In total, 14 and 20 primer pairs were selected for SSR markers of AG-2-2IIIB and AG-4HGI, respectively, which showed high amplification success and strong polymorphisms for genotyping the entire set of tested strains (Tables S3 and S4).

### 2.4. DNA Extraction and PCR

Genomic DNA was extracted from the mycelia of strains AG-2-2IIIB and AG-4HGI collected from each PDA-CF plate using the cetyltrimethylammonium bromide (CTAB) procedure, and the resulting DNA was used as a template for PCR [49]. For SSR genotyping, the forward primers were labeled with different fluorescent dyes (Dye set: FAM, ROX, TRMA, HEX; applied by Tsingke Biotech Co., Ltd., Beijing, China) at the 5′ end. PCR amplifications were performed in a 25 µL PCR mixture that included 1 µL DNA template (100 µg·mL^−1^), 9.5 µL ddH_2_O, 12.5 µL 2× T5 Super PCR Mix (Tsingke Biotech Co., Ltd.), and 1 µL each of the two primers (10 µM). The amplification was conducted in an Eppendorf Mastercycler^®^ using the following protocol: an initial denaturation step at 95 °C for 5 min, followed by 35 cycles of denaturation at 94 °C for 30 s; annealing at a primer-specific annealing temperature for 30 s, and extension at 72 °C for 30 s; with a final extension for 5 min at 72 °C. The obtained amplicons were then sequenced using an ABI 3730 DNA sequencer (Applied Biosystems, Waltham, MA, USA).

### 2.5. Population Genetics

It was validated that AG-2-2IIIB and AG-4HGI were functional diploids (i.e., a dikaryons) [41,50,51]. Sequenced fragments of identical size originating from the same primer pair were considered as alleles. One or two alleles per locus were present in both the AG-2-2IIIB and AG-4HGI strains and were scored as either homozygote or heterozygote. Alleles of each strain per loci were aligned using GeneMarker v.2.2.0 software (SoftGenetics, State College, PA, USA).

Then, the genetic variation for each of the assigned geographical regions was analyzed [52,53]. Firstly, the selective neutrality of all the SSR markers was evaluated using the Ewens–Watterson test via POPGENE v. 1.32 [42]. Then, the population genetic diversity data were statistically analyzed using POPGENE v. 1.32 according to the method of Meng et al. [54]. The data included the number of alleles (*Na*), number of effective alleles (*Ne*), observed heterozygosity (*Ho*), expected heterozygosity (*He*), heterozygosity (*H*), inbreeding coefficient (*Fis*), population differentiation index (*Fst*), and the gene flow (*Nm*) at each SSR locus. In addition, the data of *Na*, *Ne*, *Ho*, *He*, *H*, and Shannon index (*I*) for each population, Nei’s genetic similarity and genetic distance among populations were also calculated using POPGENE v. 1.32 [52,53,55]. Finally, phylogenetic trees of each population were constructed with SHAN program in NTSYSpc v. 2.1, applying the unweighted pair-group method with arithmetic average (UPGMA) based on genetic similarity to analyze the genetic diversity among the populations [56].

### 2.6. Population Structure

The population structure of strains AG-2-2IIIB and AG-4HGI was analyzed using the STRUCTURE v. 2.3.4 software [57]. The Bayesian distinct Monte Carlo Markov Chain (MCMC) approach was implemented through the same software, using the protocol described by Tsui et al. [58]. A 100,000 burn-in period followed by 1,000,000 iterations was implemented using an admixture model, and the correlated allele frequencies for K-values were between 1 and 10. For each simulated cluster for K = 1–10, ten runs were repeated independently for consistency [58]. Then, the correlated allele frequencies for K-values were calculated using Structure Harvester to estimate the optimal K-value [59]. Finally, the replicate simulations of cluster membership (q-matrices) at the optimal K-value were used as input for CLUMPP_Windows v. 1.1.2 [60] using the Fullsearch algorithm, with weighted H and the G similarity statistic. Summarized cluster membership matrices (*q*-values) for both individuals and populations were visualized using DISTRUCT v. 1.1 [61].

Nei’s unbiased genetic distance among all pairs of sampling populations was calculated with GenALEx v. 6.5 and visualized through Principal Coordinates Analysis (PCoA) [62,63]. The historical migration rate (*M*) and number of valid individuals among the geographic regions were evaluated using MIGRATE v. 3.6.11 [64].

## 3. Results

### 3.1. Transcriptome Sequencing and Analysis

After stringent quality assessment, 7.81 Gb and 8.49 Gb of clean transcriptome-sequencing data were obtained from AG-2-2IIIB and AG-4HGI, respectively. The QualityScore 30 (Q30) values obtained from AG-2-2IIIB and AG-4HGI were 93.00% and 92.26%, respectively. Based on the clean reads, 23,240 and 31,035 unigenes were assembled, of which 8218 and 8308 were longer than 1000 bp, respectively.

### 3.2. SSR Marker Development of Strains AG-2-2IIIB and AG-4HGI

Based on the unigene library, the SSR identification was analyzed, and 1232 SSR loci were obtained in total, with 592 from AG-2-2IIIB and 640 from AG-4HGI (Table 1). The mono-nucleotide repeat motifs were the most abundant in strains AG-2-2IIIB and AG-4HGI, with the number of loci being 390 (65.88%) and 431 (67.34%), respectively, followed by tri-nucleotide repeat motifs (111, 116), di-nucleotide repeat motifs (65, 66), hexa-nucleotide repeat motifs (18, 18), tetra-nucleotide repeat motifs (7, 7), and penta-nucleotide repeat motifs (1, 2) (Table 1). Based on the presence of different motifs, 14 and 20 polymorphic SSR loci were selected for the population genetic structure analysis of the AG-2-2IIIB and AG-4HGI strains, respectively.

### 3.3. Genetic Variation and Diversity

Fourteen and twenty SSR loci were used to analyze the genetic structure of strains AG-2-2IIIB and AG-4HGI, respectively. All the primers designed for each SSR of strains AG-2-2IIIB and AG-4HGI provided distinct amplicons of the expected size. The amplicon size of strains AG-2-2IIIB ranged from 138 bp to 301 bp (Table S5), while the amplicon size of strains AG-4HGI ranged from 133 bp to 315 bp (Table S6). The observed fixation indexes had a 95% confidence interval for the analysis of selected neutrality of the SSR loci from AG-2-2IIIB and AG-4HGI, suggesting that all SSR loci conformed to neutral expectation (Tables S5 and S6).

The genetic diversity of 134 strains of AG-2-2IIIB from 3 populations was determined using 14 SSR loci (Table 2). The number of alleles of each locus was determined, which ranged from 5 to 14 (with an average of 8.0714). Then, the *Ne*, *Ho*, and *He* of each locus were calculated: *Ne* ranged from 1.1835 to 6.2926 (with an average of 3.0720), *Ho* ranged from 0.1343 to 0.7836 (with an average of 0.5159), and *He* ranged from 0.1556 to 0.8443 (with an average of 0.6012). The *Ho* of each locus was lower than the respective *He*, which implied that inbreeding existed in the AG-2-2IIIB population. The *H* of the 14 loci ranged from 0.1550 to 0.8411 (with an average of 0.5989). Deviating from 0, the *Fis* ranged from −0.0746 to 0.2671 (with an average of 0.0595), which implied that there was a considerable degree of inbreeding in the populations. Furthermore, the *Fis* of five loci was negative, which indicated that an excess of heterozygotes existed in these loci. The *Fst* ranged from 0.0168 to 0.0608 (with an average of 0.0608). Except for the loci C8837 (0.0598) and C14161 (0.0608), the *Fst* was less than 0.05, which indicated that the differentiation in the AG-2-2IIIB population was relatively weak. The *Nm* ranged from 3.8640 to 14.6695 (with an average of 6.0336). Except for loci C8837 (3.9325) and C14161 (3.8640), all the *Nm* were above four, suggesting a random mating among individuals within the population.

The genetic diversity of 145 strains of AG-4HGI from 3 populations was determined using 20 SSR markers (Table 3). The number of alleles of each locus was identified, which ranged from 3 to 12 (with an average of 5.5600). Then, the *Ne*, *Ho*, and *He* of each locus were calculated: *Ne* ranged from 1.0352 to 4.8551 (with an average of 2.3926), *Ho* ranged from 0.0345 to 0.9586 (with an average of 0.5807), and *He* ranged from 0.0341 to 0.7968 (with an average of 0.4936). The *Ho* of each locus was higher than the respective *He*, which implied that either a founder effect (genetic drift) or the bottleneck effect took place in the AG-4HGI population. The *H* of the 20 loci ranged from 0.0340 to 0.7940 (with an average of 0.4917). Deviating from 0, the *Fis* ranged from −0.8618 to 0.3669 (−0.2537), which implied that there was a considerable degree of inbreeding in the populations. Furthermore, the *Fis* of sixteen loci was negative, which indicated that an excess of heterozygotes existed in these loci. The *Fst* ranged from 0.0116 to 0.1128 (with an average of 0.0421). The differentiation in the population was relatively weak, for the *Fst* of more than half of the loci was less than 0.05. The *Nm* ranged from 1.9664 to 33.5499 (with an average of 5.6837). The average *Nm* of the 20 loci was above 4, indicating random mating among individuals within the population.

As previously stated, the sampling locations of AG-2-2IIIB and AG-4HGI were grouped into three populations based on the geography, climate, and agricultural practices: Northeast China (NE), Northern China (NC), and Northwest China (NW). The genetic diversity analysis among the three populations showed only minor differences among the different populations in both AG-2-2IIIB and AG-4HGI (Table 4). For the AG-2-2IIIB strains, the highest gene diversity was found in the NE population (0.5833), followed by NC (0.5806) and NW (0.5582). The *Ne* in the NC population was the highest (2.8814), while the *He* in the NE population was the highest (0.5879). The *I* of the three populations of AG-2-2IIIB strains showed that the genetic diversity in NC was the highest, followed by NE and NW. Interestingly, for the AG-4HGI strains, the highest gene diversity was in the NW population (0.5120), followed by NE (0.4730) and NC (0.4227). Both the *Ne* (2.4521) and *He* (0.5186) in the NW population were the highest. The *I* value of the three populations of AG-4HGI strains showed that the genetic diversity in the NW population was the highest, followed by NE and NC.

**Figure 2:**
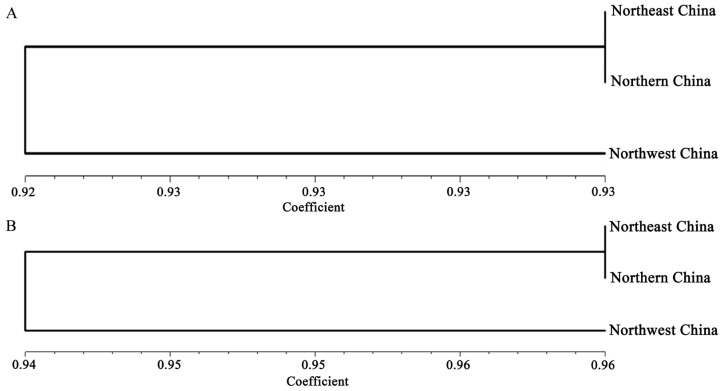
Phylogenetic trees of *Rhizoctonia solani* AG-2-2IIIB and AG-4HGI strains using unweighted pair-group method arithmetic means (UPGMA). The basal node represents the bootstrap from 1000 replicate trees. Genetic distance was calculated based on the method of Nei. (**A**): Phylogenetic tree of *R. solani* AG-2-2IIIB. (**B**): Phylogenetic tree of *R. solani* AG-4HGI.

Based on the genetic similarity between the populations, phylogenetic trees of each population were constructed with the NTSYSpc v. 2.1 software (Figure 2). For AG-2-2IIIB and AG-4HGI strains, the NE population showed a closer genetic relationship with the NC population, and clustered in a clade, which implied a close exchange of the AG-2-2IIIB and AG-4HGI strains in these two populations.

### 3.4. Population Structure and Differentiation

**Figure 3:**
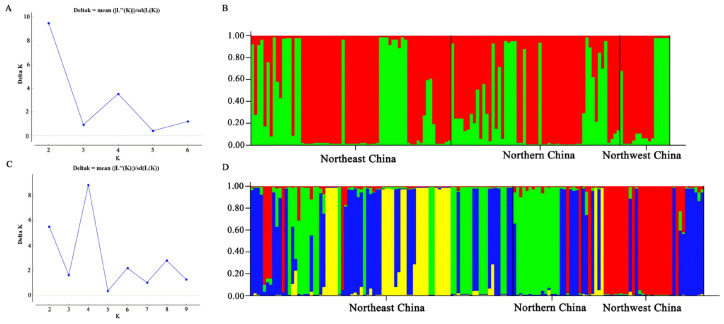
Population structure of AG-2-2IIIB and AG-4HGI. Different shadings represent different genetic groups; each column represents an individual strain, and the height of the column segments shows the probability of assigning this strain to a particular genetic group. The height of each shaded region within an individual bar is the measure of proportional affiliation. (**A**): Plot of K against delta K for AG-2-2IIIB. (**B**): Population structure of AG-2-2IIIB based on 14 loci, when K = 2; *q*1 red, *q*2 green. (**C**): Plot of K against delta K for AG-4HGI. (**D**): Population structure of AG-4HGI based on 20 loci, when K = 4; *q*1 red, *q*2 green, *q*3 yellow, and *q*4 blue.

The Bayesian cluster analysis of AG-2-2IIIB and AG-4HGI strains was conducted using STRUCTURE v. 2.3.4. The results indicated that the number of genetically distinct ancestral populations was best represented by K = 2 and 4 clusters (Figure 3A,C), which were the highest values of ΔK for AG-2-2IIIB and AG-4HGI strains, respectively.

All the three populations of AG-2-2IIIB strains were assigned to clusters *q*1 (red) and *q*2 (green), with *q*1 being the dominant genetic group (Figure 3B), suggesting that gene flow existed among the populations. The populations of AG-4HGI strains were assigned to four clusters, *q*1 (red), *q*2 (green), *q*3 (yellow), and *q*4 (blue) (Figure 3D), indicating that gene flow existed among the population groups. However, the dominant genetic group was different in the three populations. For the NE population, the dominant genetic group was *q*4, followed by *q*3, *q*2, and *q*1. For the NC population, the dominant genetic group was *q*2, followed by *q*4, *q*1, and *q*3. For the NW population, the dominant genetic group was *q*1, followed by *q*4, *q*3, and *q*2. The differentiation of different populations of AG-4HGI strains indicated that even though gene exchange occurred among the three populations, geographical isolation was still present.

**Figure 4:**
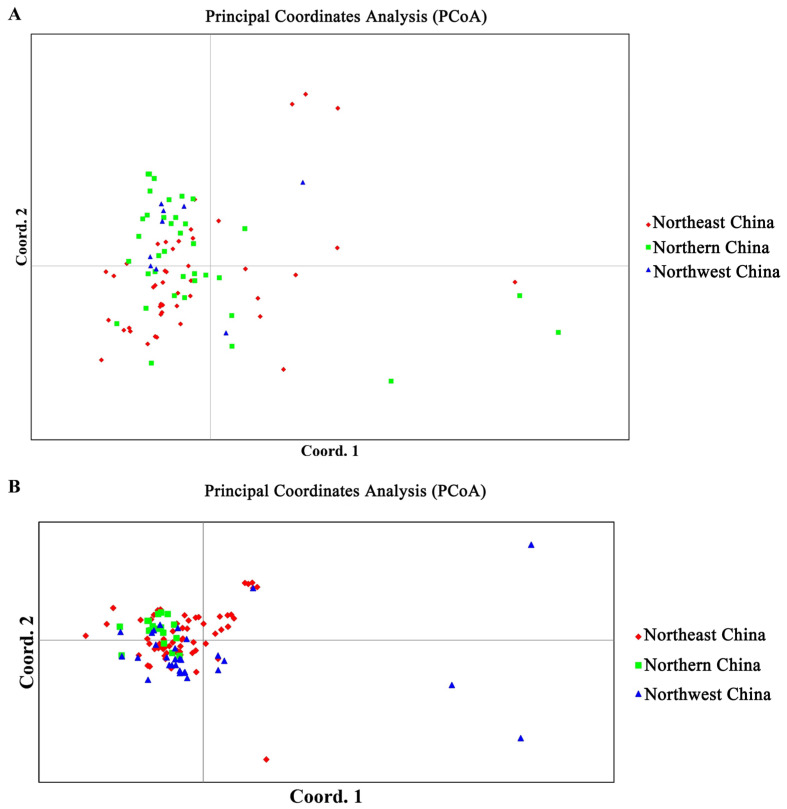
Principal Coordinates Analysis (PCoA) among three populations based on Nei’s genetic distance using GenAlEx. (**A**): PCoA of AG-2-2IIIB populations. (**B**): PCoA of AG-4HGI populations.

The PCoA was conducted on AG-2-2IIIB and AG-4HGI strains from three geographical populations (Figure 4). Both strains were not clearly separated into three groups, and the strains from the three populations were mainly clustered within the two left quadrants of the PCoA (Figure 4). The PCoA results suggest that gene exchange occurred among the three populations of AG-2-2IIIB and AG-4HGI strains, which is similar to the results obtained from the STRUCTURE analysis.

Considerable levels of gene flow were observed among the populations with an estimated number of historical migration rate (*M*) (Table 5). For the AG2-2IIIB strains, the historical migration rate from North China to Northeast China was the highest (33.667), followed by that from Northeast China to North China (32.33), while the historical migration rate from Northwest China to North China was the lowest (15.667). However, for AG-4HGI strains, the highest historical migration rate from Northwest China to North China was the highest (311.340), followed by that from Northeast China to Northwest China (30.695), while the historical migration rate from North China to Northwest China was the lowest (8.073). In addition, the observed gene flow was asymmetric between these three populations both in AG-2-2IIIB and AG-4HGI strains (Table 5).

## 4. Discussion

Clarifying the genetic relationships and population dynamics of species from different geographic areas is crucial to elucidate the effects of epidemiology on plant diseases [35,65]. In this study, we analyzed the microsatellite loci in the transcriptome of AG-2-2IIIB and AG-4HGI, which were the main pathogens causing seedling damping-off and crown and root rot disease in sugar beets [15,16,17,18,19]. Then, 14 and 20 polymorphic microsatellite loci were selected to analyze the population genetic structures of AG-2-2IIIB and AG-4HGI, respectively. The results suggested that the individuals from both AG-2-2IIIB and AG-4HGI populations mated randomly, and a considerable degree of inbreeding took place. In addition, differentiation was found among the different populations of AG-4HGI, suggesting that either a founder effect (genetic drift) or a bottleneck effect could have occurred. Therefore, this set of microsatellite markers may be an important tool for population genetic investigations on these two AGs of *Rhizoctonia*.

SSR has been widely used for analyzing the reproductive mode, geographic migration, and relationships with growth rate or fungicide sensitivity of *Rhizoctonia*, including *R. solani* AG-1 IA [33,66,67,68], AG-3 PT [34], AG-4HGI [41], and *R. cerealis* [39,69]. In this study, the population genetic structure of *R. solani* AG-2-2 IIIB and AG-4HGI was analyzed, and the result suggested that the individuals in AG-2-2IIIB and AG-4HGI populations in China mated randomly, and asymmetric gene flow were observed among the populations with an estimated number of historical migration rate. Similarly, an investigation into the genetic structure of *R. solani* AG-1 IA populations with SSR markers causing soybean foliar blight also indicated asymmetric geographical migration within Brazil [68].

Transcriptome sequencing is the most widely used method for SSR markers development because it is highly efficient, cost-effective, and rapid [70,71]. In our previous study, a total of 1140 simple sequence repeats were identified from *Alternaria tenuissima*, a pathogenic fungus causing foliar disease in tomato, and its population genetics were identified in four regions within China [42]. In this study, a total of 592 and 640 SSR loci were identified from AG-2-2IIIB and AG-4HGI, respectively. The repeat motifs included mono-nucleotide, di-nucleotide, tri-nucleotide, tetra-nucleotide, penta-nucleotide, and hexa-nucleotide, with mono-nucleotide repeat motifs being the most abundant in both AG-2-2IIIB and AG-4HGI strains.

Population subdivision (*Fst*) usually indicates various degrees of significant gene flow among all populations, because a higher gene exchange triggers a higher degree of genetic similarity. Gene flow tends to homogenize alleles among populations and keeps geographic populations genetically interconnected by introducing, reproducing, and ensuring the survival of the introduced organism. This causes the establishment of new alleles or genotypes in new locations and determines the relative effect of selection and genetic drift [41,42,72]. In this study, high to moderate gene flow and low to moderate population subdivision (*Fst*) were detected among populations of AG-2-2IIIB and AG-4HGI, which indicated that weak differentiation existed in the populations of AG-2-2IIIB and AG-4HGI, and the individuals within the population were randomly mated. The average observed heterozygosity of AG-2-2IIIB was lower than the expected heterozygosity, suggesting that inbreeding existed within the population of AG-2-2IIIB. Interestingly, the average observed heterozygosity of AG-4HGI was higher than the expected heterozygosity. It is therefore possible that the AG-4HGI population could have experienced either a founder effect (genetic drift) or a bottleneck effect. Both human and natural factors can lead to the occurrence of genetic drift, migration, gene recombination, natural selection, and mutation, all of which cause genetic variation within species and also serve as the main driving force for species evolution [73,74]. The founder effect (genetic drift) or the bottleneck effect in the AG-4HGI population may have been caused by the use of fungicides during this period, which would have caused the death of some individuals, or by the change in rotation crops, which would have introduced new pathogens.

The three populations of AG-2-2IIIB and AG-4HGI were grouped based on the geography, climate, agricultural practices, and the sampling locations. Only minor differences were observed in the genetic diversity among the three populations of both strains. The cluster analysis revealed that the genetic relationship of the Northeast China population and Northern China population was much closer than that of Northwest China population, which indicated that the strains of AG-2-2IIIB or AG-4HGI from Northeast China population and Northern China population communicated more frequently. It was previously reported that the genetic differentiation in AG-3 PT from different geographical populations was related to the frequency of communication between regions [75]. Geographically, the areas of sugar beet cultivation from which the Northeast China and Northern China populations were sampled are relatively closer, which encouraged communication between these two strain populations, resulting in a lower level of genetic differentiation.

Population structure and PCoA also revealed that the genetic structures of the three populations of AG-2-2IIIB and AG-4HGI were highly admixed and could not be separated into three distinct major clusters, which is incoordinate with the gene flow results. Among the AG-2-2IIIB population, the historical migration rate between North China and Northeast China was the highest, while the highest historical migration rate for the AG-4HGI population was from Northeast China to North China, followed by from Northeast China to Northwest China. In addition, the population composition of AG-4HGI was significantly more diverse than that of AG-2-2IIIB, and differentiation was also observed among the different populations of AG-4HGI. The AG-2-2IIIB strain mainly infects sugar beet, causing sugar beet seedling damping-off and crown and root rot disease [15,19], and is occasionally isolated from diseased potatoes [76]. Coincidentally, when sugar beet was introduced to China on a large scale, the first beet sugar factory was built in Acheng district, Harbin, Heilongjiang province, Northeast China, then expanded to the North China and Northwest China [77]. Therefore, human-mediated factors such as seed transport and the movement of harvesting machinery during the beet cultivation process may have facilitated gene exchange and historical migration of AG-2-2IIIB. However, AG-4HGI has a wide range of hosts, such as maize [20], beans [78], and potato [13], which are commonly rotated or inter-cropped with sugar beet in these regions. Additionally, the rotation crops in different geographic regions were not the same, which led to a differentiation among the different populations of AG-4HGI. Furthermore, it was reported that AG-4 had a bipolar heterothallic mating system controlled by a single genetic factor with multiple alleles, and basidiospores may have important ecological and population consequences [41]. The observed high genetic diversity may be related to the recombination caused by sexual reproduction.

## Figures and Tables

**Table 1:** Loci of simple sequence repeats (SSRs) developed from strains AG-2-2IIIB and AG-4HGI of *Rhizoctonia solani*.

Type of Repeat Motifs	AG-2-2IIIB		AG-4HGI	
	Number of Loci	Ratio (%)	Number of Loci	Ratio (%)
Mono-nucleotide	390	65.88	431	67.34
Di-nucleotide	65	10.98	66	10.31
Tri--nucleotide	111	18.75	116	18.12
Tetra-nucleotide	7	1.18	7	1.09
Penta-nucleotide	1	0.17	2	0.31
Hexa-nucleotide	18	3.05	18	2.81
Total	592	100	640	100

**Table 2:** Genetic diversity of *Rhizoctonia solani* AG-2-2IIIB.

Loci	*Na* ^a^	*Ne* ^b^	*I* ^c^	*Ho* ^d^	*He* ^e^	*H* ^f^	*Fis* ^g^	*Fst* ^h^	*Nm* ^i^
C6248	7	2.3127	1.2006	0.5746	0.5697	0.5676	−0.0536	0.0497	4.7803
C14525	8	3.2047	1.4015	0.4701	0.6905	0.6880	0.2595	0.0455	5.2447
C8703	8	4.6956	1.7251	0.7836	0.7900	0.7870	−0.1240	0.0366	6.5753
C7683	7	2.3966	1.1423	0.6015	0.5849	0.5827	−0.0951	0.0371	6.4851
C15210	6	2.6390	1.1056	0.5970	0.6234	0.6211	0.1021	0.0242	10.0700
C8837	14	6.2926	2.1169	0.4851	0.8442	0.8411	0.2671	0.0598	3.9325
C14161	9	3.5253	1.5291	0.6791	0.7190	0.7163	−0.0746	0.0608	3.8640
C9144	5	1.1835	0.3784	0.1343	0.1556	0.1550	0.1160	0.0361	6.6695
C12183	11	4.7481	1.7207	0.6767	0.7924	0.7894	0.1011	0.0433	5.5269
C15253	6	1.2642	0.4850	0.1818	0.2098	0.2090	0.0476	0.0168	14.6695
C13740	6	3.2814	1.3461	0.5076	0.6979	0.6952	0.0811	0.0294	8.2533
C9782	11	2.6992	1.4274	0.5714	0.6319	0.6295	−0.0039	0.0383	6.2761
C4407	6	1.7783	0.8948	0.3955	0.4393	0.4377	0.0117	0.0336	7.1833
C14499	9	2.9875	1.3659	0.5639	0.6678	0.6653	0.1552	0.0184	13.3346
Average	8.0714	3.0720	1.2742	0.5159	0.6012	0.5989	0.0595	0.0398	6.0336

^a^ *Na*, number of alleles. ^b^ *Ne*, number of effective alleles. ^c^ *I*, Shannon index. ^d^ *Ho*, observed heterozygosity. ^e^ *He*, expected heterozygosity. ^f^ *H*, heterozygosity. ^g^ *Fis*, inbreeding coefficient. ^h^ *Fst*, population differentiation index. ^i^ *Nm*, gene flow.

**Table 3:** Genetic diversity of *Rhizoctonia solani* AG-4HGI.

Loci	*Na* ^a^	*Ne* ^b^	*I* ^c^	*Ho* ^d^	*He* ^e^	*H* ^f^	*Fis* ^g^	*Fst* ^h^	*Nm* ^i^
012785	5	2.6488	1.0636	0.7310	0.6246	0.6225	−0.1556	0.0339	7.1325
004329	2	1.1870	0.2937	0.1724	0.1581	0.1576	−0.2006	0.0437	5.4763
015286	4	1.4337	0.5808	0.3241	0.3035	0.3025	−0.1309	0.0669	3.4895
023115	10	4.8551	1.8099	0.7724	0.7968	0.7940	−0.1234	0.1128	1.9664
057704	7	2.3531	1.1579	0.6966	0.5770	0.5750	−0.2723	0.0509	4.6643
006128	4	1.9638	0.7181	0.7655	0.4925	0.4908	−0.6914	0.0399	6.0214
007713	5	1.3809	0.5919	0.3103	0.2768	0.2758	−0.1539	0.0190	12.8991
010525	3	1.0642	0.1491	0.0483	0.0605	0.0603	0.0311	0.0242	10.064
004651	4	2.8586	1.1427	0.8759	0.6524	0.6502	−0.4311	0.0665	3.5099
063922	10	2.9201	1.3813	0.7241	0.6598	0.6576	−0.3281	0.0619	3.7898
016188	10	3.0320	1.4966	0.8889	0.6725	0.6702	−0.4461	0.0826	2.7753
068450	12	3.0333	1.5410	0.8897	0.6726	0.6703	−0.3544	0.0587	4.0076
012305	4	2.2649	0.9636	0.6621	0.5604	0.5585	−0.2073	0.0875	2.6068
013519	6	3.9915	1.5093	0.8828	0.7521	0.7495	−0.3727	0.1029	2.1784
005937	3	2.0554	0.7563	0.9586	0.5153	0.5135	−0.8618	0.0074	33.5499
060842	3	1.1323	0.2459	0.0621	0.1172	0.1168	0.3669	0.0214	11.452
042482	4	2.4738	1.0239	0.4414	0.5978	0.5958	0.0640	0.0985	2.2876
022653	5	2.6636	1.1164	0.4552	0.6267	0.6246	0.0695	0.0826	2.7752
058474	9	3.5042	1.5538	0.9172	0.7171	0.7146	−0.5111	0.0447	5.3402
011009	3	1.0352	0.0957	0.0345	0.0341	0.0340	−0.0214	0.0116	21.3258
Average	5.6500	2.3926	0.9596	0.5807	0.4936	0.4917	−0.2537	0.0421	5.6837

^a^ *Na*, number of alleles. ^b^ *Ne*, number of effective alleles. ^c^ *I*, Shannon index. ^d^ *Ho*, observed heterozygosity. ^e^ *He*, expected heterozygosity. ^f^ *H*, heterozygosity. ^g^ *Fis*, inbreeding coefficient. ^h^ *Fst*, population differentiation index. ^i^ *Nm*, gene flow.

**Table 4:** Genetic diversity in three geographic regions of *Rhizoctonia solani* AG-2-2IIIB and AG-4HGI strains sampled from the main sugar beet production regions in China.

Anastomosis Group	Population ^a^	Number of Strains	*Na* ^b^	*Ne* ^c^	*I* ^d^	*Ho* ^e^	*He* ^f^	*H* ^g^
AG-2-2IIIB	NE	64	6.7857	2.8636	1.1943	0.4798	0.5879	0.5833
	NC	54	6.5000	2.8814	1.2040	0.5325	0.5861	0.5806
	NW	16	3.7857	2.7571	1.0396	0.6071	0.5763	0.5582
AG-4HGI	NE	84	5.0000	2.2753	0.9080	0.5630	0.4758	0.4730
	NC	22	3.0500	2.0418	0.7388	0.5864	0.4325	0.4227
	NW	39	4.3500	2.4521	0.9532	0.6154	0.5186	0.5120

^a^ NE, Northeast China; NC, Northern China; NW, Northwest China. ^b^ *Na*, number of alleles. ^c^ *Ne*, number of effective alleles. ^d^ *I*, Shannon index. ^e^ *Ho*, observed heterozygosity. ^f^ *He*, expected heterozygosity. ^g^ *H*, heterozygosity.

**Table 5:** Evaluation of the average mutation rate (*θ*) and historical migration rate (*M*) of the *Rhizoctonia solani* AG-2-2IIIB and AG-4HGI population.

Anastomosis Group	Population ^a^	Average Mutation Rate (*θ*)	Historical Migration Rate (*M*)		
			NE	NC	NW
AG-2-2IIIB	NE	0.0965	-	32.333	31.000
	NC	0.0965	33.667	-	21.667
	NW	0.0898	18.333	15.667	-
AG-4HGI	NE	0.0951	-	16.726	30.695
	NC	0.0381	23.708	-	8.073
	NW	0.0930	11.864	311.340	-

^a^ NE, Northeast China; NC, Northern China; NW, Northwest China.

## Data Availability

The original contributions presented in this study are included in the article and Supplementary Materials. Further inquiries can be directed to the corresponding author.

## Supplementary Materials

The following supporting information can be downloaded at: https://www.mdpi.com/article/10.3390/jof12020097/s1. Table S1. Information of the *Rhizoctonia solani* AG-2-2IIIB strains used for simple sequence repeats (SSRs) analysis. Table S2. Information of the *Rhizoctonia solani* AG-4HGI strains used for simple sequence repeats (SSRs) analysis. Table S3. Repeat motifs and primer sequences of the 14 loci of simple sequence repeats (SSRs) from *Rhizoctonia solani* AG-2-2IIIB. Table S4. Repeat motifs and primer sequences of the 20 loci of simple sequence repeats (SSRs) from *Rhizoctonia solani* AG-4HGI. Table S5. The size of amplicons, number of strains, and selective neutrality test of *Rhizoctonia solani* AG-2-2IIIB using fourteen markers of simple sequence repeats (SSRs). Table S6. The size of amplicon, number of strains, and selective neutrality test of *Rhizoctonia solani* AG-4HGI using twenty markers of simple sequence repeats (SSRs).
