# Supplementary material for: Genetic Structure of Populations of Rhizoctonia solani Anastomosis Group (AG)-2-2IIIB and AG-4HGI Causing Sugar Beet Root Diseases in China
Source: J Fungi (Basel). 2026 Jan 30;12(2):97. doi: 10.3390/jof12020097 (PMC12941418; doi:10.3390/jof12020097)
Supplement: Supplementary file 1 [file jof-12-00097-s001.zip › Table S1.pdf]

Table S1. Information of the *Rhizoctonia solani* AG-2-2IIIB strains used for simple sequence repeats (SSRs) analysis.

Strain code	Sampling origin	Sampling year	Population [a]
R5	Heihe city, Heilongjiang province	2009	NE
R6	Heihe city, Heilongjiang province	2009	NE
R8	Suihua city, Heilongjiang province	2009	NE
R19	Chifeng city, Inner Mongolia autonomous region	2009	NE
R10	Ulanqab city, Inner Mongolia autonomous region	2009	NC
R11	Ulanqab city, Inner Mongolia autonomous region	2009	NC
R12	Ulanqab city, Inner Mongolia autonomous region	2009	NC
R13	Ulanqab city, Inner Mongolia autonomous region	2009	NC
R15	Ulanqab city, Inner Mongolia autonomous region	2009	NC
R16	Ulanqab city, Inner Mongolia autonomous region	2009	NC
R21	Ulanqab city, Inner Mongolia autonomous region	2009	NC
R9	Ulanqab city, Inner Mongolia autonomous region	2009	NC
R25	Qiqihar city, Heilongjiang province	2010	NE
R38	Suihua city, Heilongjiang province	2010	NE
R42	Chifeng city, Inner Mongolia autonomous region	2010	NE
R28	Ulanqab city, Inner Mongolia autonomous region	2010	NC
R29	Ulanqab city, Inner Mongolia autonomous region	2010	NC
R30	Ulanqab city, Inner Mongolia autonomous region	2010	NC
R41	Baotou city, Inner Mongolia autonomous region	2010	NC
R32	Datong city, Shanxi province	2010	NC
R33	Datong city, Shanxi province	2010	NC
R36	Changji city, Xinjiang Uygur autonomous region	2010	NW
RR13	Changchun city, Jilin province	2011	NE
RR21	Changchun city, Jilin province	2011	NE
RR11	Ulanqab city, Inner Mongolia autonomous region	2011	NC
RR16	Ulanqab city, Inner Mongolia autonomous region	2011	NC
RR17	Ulanqab city, Inner Mongolia autonomous region	2011	NC
RR25	Ulanqab city, Inner Mongolia autonomous region	2011	NC
R2(12)	Qiqihar city, Heilongjiang province	2012	NE
R4(12)	Qiqihar city, Heilongjiang province	2012	NE
JL-2	Changchun city, Jilin province	2013	NE
RNM1	Ulanqab city, Inner Mongolia autonomous region	2013	NC
RNM3	Ulanqab city, Inner Mongolia autonomous region	2013	NC
RNM4	Ulanqab city, Inner Mongolia autonomous region	2013	NC
RNM5	Ulanqab city, Inner Mongolia autonomous region	2013	NC
RNM7	Ulanqab city, Inner Mongolia autonomous region	2013	NC
RNM8	Ulanqab city, Inner Mongolia autonomous region	2013	NC
RNM9	Ulanqab city, Inner Mongolia autonomous region	2013	NC
RN10	Chifeng city, Inner Mongolia autonomous region	2014	NE
RN6	Ulanqab city, Inner Mongolia autonomous region	2014	NC
RN100	Chifeng city, Inner Mongolia autonomous region	2015	NE

RN102	Chifeng city, Inner Mongolia autonomous region	2015	NE
RN104	Chifeng city, Inner Mongolia autonomous region	2015	NE
RN78	Chifeng city, Inner Mongolia autonomous region	2015	NE
RN91	Chifeng city, Inner Mongolia autonomous region	2015	NE
RN92	Chifeng city, Inner Mongolia autonomous region	2015	NE
RN94	Chifeng city, Inner Mongolia autonomous region	2015	NE
RN95	Chifeng city, Inner Mongolia autonomous region	2015	NE
RN97	Chifeng city, Inner Mongolia autonomous region	2015	NE
RN98	Chifeng city, Inner Mongolia autonomous region	2015	NE
RN99	Chifeng city, Inner Mongolia autonomous region	2015	NE
RN28	Hinggan league, Inner Mongolia autonomous region	2015	NE
RN29	Hinggan league, Inner Mongolia autonomous region	2015	NE
RN31	Hinggan league, Inner Mongolia autonomous region	2015	NE
RN39	Hinggan league, Inner Mongolia autonomous region	2015	NE
RN43	Hinggan league, Inner Mongolia autonomous region	2015	NE
RN50	Hinggan league, Inner Mongolia autonomous region	2015	NE
RN53	Hinggan league, Inner Mongolia autonomous region	2015	NE
RN56	Hinggan league, Inner Mongolia autonomous region	2015	NE
RN57	Hinggan league, Inner Mongolia autonomous region	2015	NE
RN60	Hinggan league, Inner Mongolia autonomous region	2015	NE
RN61	Hinggan league, Inner Mongolia autonomous region	2015	NE
RN62	Hinggan league, Inner Mongolia autonomous region	2015	NE
RN63	Hinggan league, Inner Mongolia autonomous region	2015	NE
RN66	Hinggan league, Inner Mongolia autonomous region	2015	NE
RN67	Hinggan league, Inner Mongolia autonomous region	2015	NE
RN69	Hinggan league, Inner Mongolia autonomous region	2015	NE
RN72	Hinggan league, Inner Mongolia autonomous region	2015	NE
RN74	Hinggan league, Inner Mongolia autonomous region	2015	NE
RN75	Hinggan league, Inner Mongolia autonomous region	2015	NE
RN76	Hinggan league, Inner Mongolia autonomous region	2015	NE
RN14	Ulanqab city, Inner Mongolia autonomous region	2015	NC
RN15	Ulanqab city, Inner Mongolia autonomous region	2015	NC
RX28	Ili city, Xinjiang Uygur autonomous region	2015	NW
RX29	Ili city, Xinjiang Uygur autonomous region	2015	NW
RN124	Chifeng city, Inner Mongolia autonomous region	2016	NE
RN125	Chifeng city, Inner Mongolia autonomous region	2016	NE
RN126	Chifeng city, Inner Mongolia autonomous region	2016	NE
RN127	Chifeng city, Inner Mongolia autonomous region	2016	NE
RN128	Chifeng city, Inner Mongolia autonomous region	2016	NE
RN129	Chifeng city, Inner Mongolia autonomous region	2016	NE
RN130	Chifeng city, Inner Mongolia autonomous region	2016	NE
RN131	Chifeng city, Inner Mongolia autonomous region	2016	NE
RN132	Chifeng city, Inner Mongolia autonomous region	2016	NE
RN144	Chifeng city, Inner Mongolia autonomous region	2016	NE

RN163	Chifeng city, Inner Mongolia autonomous region	2016	NE
RN164	Chifeng city, Inner Mongolia autonomous region	2016	NE
RN165	Chifeng city, Inner Mongolia autonomous region	2016	NE
RN166	Chifeng city, Inner Mongolia autonomous region	2016	NE
RN114	Ulanqab city, Inner Mongolia autonomous region	2016	NC
RN115	Ulanqab city, Inner Mongolia autonomous region	2016	NC
RN116	Ulanqab city, Inner Mongolia autonomous region	2016	NC
RN117	Ulanqab city, Inner Mongolia autonomous region	2016	NC
RN118	Ulanqab city, Inner Mongolia autonomous region	2016	NC
RN119	Ulanqab city, Inner Mongolia autonomous region	2016	NC
RN120	Ulanqab city, Inner Mongolia autonomous region	2016	NC
RN121	Ulanqab city, Inner Mongolia autonomous region	2016	NC
RN122	Ulanqab city, Inner Mongolia autonomous region	2016	NC
RN146	Ulanqab city, Inner Mongolia autonomous region	2016	NC
RN147	Ulanqab city, Inner Mongolia autonomous region	2016	NC
RN148	Ulanqab city, Inner Mongolia autonomous region	2016	NC
RN149	Ulanqab city, Inner Mongolia autonomous region	2016	NC
RN150	Ulanqab city, Inner Mongolia autonomous region	2016	NC
RN151	Ulanqab city, Inner Mongolia autonomous region	2016	NC
RN152	Ulanqab city, Inner Mongolia autonomous region	2016	NC
RN153	Ulanqab city, Inner Mongolia autonomous region	2016	NC
RN154	Ulanqab city, Inner Mongolia autonomous region	2016	NC
RN155	Ulanqab city, Inner Mongolia autonomous region	2016	NC
RN156	Ulanqab city, Inner Mongolia autonomous region	2016	NC
RN157	Ulanqab city, Inner Mongolia autonomous region	2016	NC
RN158	Ulanqab city, Inner Mongolia autonomous region	2016	NC
RN159	Ulanqab city, Inner Mongolia autonomous region	2016	NC
RN160	Ulanqab city, Inner Mongolia autonomous region	2016	NC
RN161	Ulanqab city, Inner Mongolia autonomous region	2016	NC
RN162	Ulanqab city, Inner Mongolia autonomous region	2016	NC
RX46	Changji city, Xinjiang Uygur autonomous region	2016	NW
RX47	Changji city, Xinjiang Uygur autonomous region	2016	NW
RX48	Changji city, Xinjiang Uygur autonomous region	2016	NW
RX49	Changji city, Xinjiang Uygur autonomous region	2016	NW
RHL19	Qiqihar city, Heilongjiang province	2017	NE
RHL20	Qiqihar city, Heilongjiang province	2017	NE
RHL21	Qiqihar city, Heilongjiang province	2017	NE
RHL24	Qiqihar city, Heilongjiang province	2017	NE
RHL25	Qiqihar city, Heilongjiang province	2017	NE
RHL26	Qiqihar city, Heilongjiang province	2017	NE
RX50	Ili city, Xinjiang Uygur autonomous region	2017	NW
RX51	Ili city, Xinjiang Uygur autonomous region	2017	NW
RX52	Ili city, Xinjiang Uygur autonomous region	2017	NW
RX53	Ili city, Xinjiang Uygur autonomous region	2017	NW

RX54	Ili city, Xinjiang Uygur autonomous region	2017	NW
RGS1	Zhangye city, Gansu province	2017	NW
RGS2	Zhangye city, Gansu province	2017	NW
RGS3	Zhangye city, Gansu province	2017	NW
RGS4	Zhangye city, Gansu province	2017	NW

[a]NE, Northeast China (including Heilongjiang, Jilin province, Chifeng city and Hinggan league of Inner Mongolia autonomous region); NC, Northern China (including Shanxi province and Ulanqab city and Baotou city of Inner Mongolia autonomous region); NW, Northwest China (including Gansu province and Xinjiang Uygur autonomous region).
