# Supplementary material for: Genetic Structure of Populations of Rhizoctonia solani Anastomosis Group (AG)-2-2IIIB and AG-4HGI Causing Sugar Beet Root Diseases in China
Source: J Fungi (Basel). 2026 Jan 30;12(2):97. doi: 10.3390/jof12020097 (PMC12941418; doi:10.3390/jof12020097)
Supplement: Supplementary file 1 [file jof-12-00097-s001.zip › Table S2.pdf]

Table S2. Information of the *Rhizoctonia solani* AG-4HGI strains used for simple sequence repeats (SSRs) analysis.

Strain code	Sampling origin	Sampling year	Population [a]
R17	Chifeng city, Inner Mongolia autonomous region	2009	NE
R18	Chifeng city, Inner Mongolia autonomous region	2009	NE
R20	Chifeng city, Inner Mongolia autonomous region	2009	NE
R31	Chifeng city, Inner Mongolia autonomous region	2009	NE
R23	Qiqihar city, Heilongjiang province	2010	NE
R24	Qiqihar city, Heilongjiang province	2010	NE
D16	Qiqihar city, Heilongjiang province	2011	NE
D17	Qiqihar city, Heilongjiang province	2011	NE
D18	Qiqihar city, Heilongjiang province	2011	NE
D19	Qiqihar city, Heilongjiang province	2011	NE
D24	Qiqihar city, Heilongjiang province	2011	NE
D31	Qiqihar city, Heilongjiang province	2011	NE
D32	Qiqihar city, Heilongjiang province	2011	NE
D1	Chifeng city, Inner Mongolia autonomous region	2011	NE
D10	Chifeng city, Inner Mongolia autonomous region	2011	NE
D2	Chifeng city, Inner Mongolia autonomous region	2011	NE
D4	Chifeng city, Inner Mongolia autonomous region	2011	NE
D5	Chifeng city, Inner Mongolia autonomous region	2011	NE
D7	Chifeng city, Inner Mongolia autonomous region	2011	NE
D8	Chifeng city, Inner Mongolia autonomous region	2011	NE
RR6	Zhangye city, Gansu province	2011	NW
RD5	Qiqihar city, Heilongjiang province	2012	NE
RD4	Changji city, Xinjiang Uygur autonomous region	2012	NW
RHL1	Qiqihar city, Heilongjiang province	2013	NE
RHL2	Qiqihar city, Heilongjiang province	2013	NE
RHL3	Qiqihar city, Heilongjiang province	2013	NE
RHL4	Qiqihar city, Heilongjiang province	2013	NE
RHL5	Qiqihar city, Heilongjiang province	2013	NE
RHL6	Qiqihar city, Heilongjiang province	2013	NE
RSX1	Datong city, Shanxi province	2013	NC
RSX2	Datong city, Shanxi province	2013	NC
RSX3	Datong city, Shanxi province	2013	NC
RSX4	Datong city, Shanxi province	2013	NC
RSX5	Datong city, Shanxi province	2013	NC
RSX6	Datong city, Shanxi province	2013	NC
RSX7	Datong city, Shanxi province	2013	NC
RX1	Changji city, Xinjiang Uygur autonomous region	2013	NW
RX10	Changji city, Xinjiang Uygur autonomous region	2013	NW
RX3	Changji city, Xinjiang Uygur autonomous region	2013	NW
RX4	Changji city, Xinjiang Uygur autonomous region	2013	NW
RX5	Changji city, Xinjiang Uygur autonomous region	2013	NW

RX6	Changji city, Xinjiang Uygur autonomous region	2013	NW
RX8	Changji city, Xinjiang Uygur autonomous region	2013	NW
RX9	Changji city, Xinjiang Uygur autonomous region	2013	NW
XJ7	Changji city, Xinjiang Uygur autonomous region	2013	NW
RX11	Shihezi city, Xinjiang Uygur autonomous region	2013	NW
RX13	Shihezi city, Xinjiang Uygur autonomous region	2013	NW
RX14	Shihezi city, Xinjiang Uygur autonomous region	2013	NW
RX15	Shihezi city, Xinjiang Uygur autonomous region	2013	NW
RX16	Shihezi city, Xinjiang Uygur autonomous region	2013	NW
RX17	Shihezi city, Xinjiang Uygur autonomous region	2013	NW
XJ2	Ili city, Xinjiang Uygur autonomous region	2013	NW
XJ6	Ili city, Xinjiang Uygur autonomous region	2013	NW
GS-5	Zhangye city, Gansu province	2013	NW
N4	Ulanqab city, Inner Mongolia autonomous region	2014	NC
RN5	Ulanqab city, Inner Mongolia autonomous region	2014	NC
RXJ3	Shihezi city, Xinjiang Uygur autonomous region	2014	NW
X2	Urumqi city, Xinjiang Uygur autonomous region	2014	NW
X5	Urumqi city, Xinjiang Uygur autonomous region	2014	NW
HLJ-22	Qiqihar city, Heilongjiang province	2015	NE
HLJ32	Qiqihar city, Heilongjiang province	2015	NE
RHL10	Qiqihar city, Heilongjiang province	2015	NE
RHL11	Qiqihar city, Heilongjiang province	2015	NE
RHL17	Qiqihar city, Heilongjiang province	2015	NE
RHL9	Qiqihar city, Heilongjiang province	2015	NE
RN79	Chifeng city, Inner Mongolia autonomous region	2015	NE
RN80	Chifeng city, Inner Mongolia autonomous region	2015	NE
RN81	Chifeng city, Inner Mongolia autonomous region	2015	NE
RN83	Chifeng city, Inner Mongolia autonomous region	2015	NE
RN84	Chifeng city, Inner Mongolia autonomous region	2015	NE
RN85	Chifeng city, Inner Mongolia autonomous region	2015	NE
RN86	Chifeng city, Inner Mongolia autonomous region	2015	NE
RN87	Chifeng city, Inner Mongolia autonomous region	2015	NE
RN88	Chifeng city, Inner Mongolia autonomous region	2015	NE
RN89	Chifeng city, Inner Mongolia autonomous region	2015	NE
RN90	Chifeng city, Inner Mongolia autonomous region	2015	NE
RN20	Ulanqab city, Inner Mongolia autonomous region	2015	NC
RN21	Ulanqab city, Inner Mongolia autonomous region	2015	NC
RN22	Ulanqab city, Inner Mongolia autonomous region	2015	NC
RN23	Ulanqab city, Inner Mongolia autonomous region	2015	NC
RN25	Ulanqab city, Inner Mongolia autonomous region	2015	NC
RX39	Changji city, Xinjiang Uygur autonomous region	2015	NW
RX40	Changji city, Xinjiang Uygur autonomous region	2015	NW
RX41	Changji city, Xinjiang Uygur autonomous region	2015	NW
RX42	Changji city, Xinjiang Uygur autonomous region	2015	NW

RX44	Changji city, Xinjiang Uygur autonomous region	2015	NW
RX45	Changji city, Xinjiang Uygur autonomous region	2015	NW
RX18	Urumqi city, Xinjiang Uygur autonomous region	2015	NW
RX19	Urumqi city, Xinjiang Uygur autonomous region	2015	NW
RX20	Urumqi city, Xinjiang Uygur autonomous region	2015	NW
RX21	Urumqi city, Xinjiang Uygur autonomous region	2015	NW
RX22	Urumqi city, Xinjiang Uygur autonomous region	2015	NW
RX23	Urumqi city, Xinjiang Uygur autonomous region	2015	NW
RN133	Chifeng city, Inner Mongolia autonomous region	2016	NE
RN134	Chifeng city, Inner Mongolia autonomous region	2016	NE
RN135	Chifeng city, Inner Mongolia autonomous region	2016	NE
RN136	Chifeng city, Inner Mongolia autonomous region	2016	NE
RN137	Chifeng city, Inner Mongolia autonomous region	2016	NE
RN138	Chifeng city, Inner Mongolia autonomous region	2016	NE
RN139	Chifeng city, Inner Mongolia autonomous region	2016	NE
RN140	Chifeng city, Inner Mongolia autonomous region	2016	NE
RN141	Chifeng city, Inner Mongolia autonomous region	2016	NE
RN142	Chifeng city, Inner Mongolia autonomous region	2016	NE
RN143	Chifeng city, Inner Mongolia autonomous region	2016	NE
RN144	Chifeng city, Inner Mongolia autonomous region	2016	NE
RN145	Chifeng city, Inner Mongolia autonomous region	2016	NE
RN106	Ulanqab city, Inner Mongolia autonomous region	2016	NC
RN107	Ulanqab city, Inner Mongolia autonomous region	2016	NC
RN108	Ulanqab city, Inner Mongolia autonomous region	2016	NC
RN109	Ulanqab city, Inner Mongolia autonomous region	2016	NC
RN110	Ulanqab city, Inner Mongolia autonomous region	2016	NC
RN111	Ulanqab city, Inner Mongolia autonomous region	2016	NC
RN112	Ulanqab city, Inner Mongolia autonomous region	2016	NC
RN113	Ulanqab city, Inner Mongolia autonomous region	2016	NC
RHL22	Ulanqab city, Inner Mongolia autonomous region	2017	NE
RHL23	Ulanqab city, Inner Mongolia autonomous region	2017	NE
BN2	Chifeng city, Inner Mongolia autonomous region	2017	NE
BN3	Chifeng city, Inner Mongolia autonomous region	2017	NE
BN4	Chifeng city, Inner Mongolia autonomous region	2017	NE
BN5	Chifeng city, Inner Mongolia autonomous region	2017	NE
BN6	Chifeng city, Inner Mongolia autonomous region	2017	NE
RN170	Chifeng city, Inner Mongolia autonomous region	2017	NE
RN171	Chifeng city, Inner Mongolia autonomous region	2017	NE
RN172	Chifeng city, Inner Mongolia autonomous region	2017	NE
RN173	Chifeng city, Inner Mongolia autonomous region	2017	NE
RN174	Chifeng city, Inner Mongolia autonomous region	2017	NE
RN175	Chifeng city, Inner Mongolia autonomous region	2017	NE
RN176	Chifeng city, Inner Mongolia autonomous region	2017	NE
RN177	Chifeng city, Inner Mongolia autonomous region	2017	NE

RN178	Chifeng city, Inner Mongolia autonomous region	2017	NE
RN179	Chifeng city, Inner Mongolia autonomous region	2017	NE
RN180	Chifeng city, Inner Mongolia autonomous region	2017	NE
RN187	Chifeng city, Inner Mongolia autonomous region	2017	NE
RN188	Chifeng city, Inner Mongolia autonomous region	2017	NE
RN189	Chifeng city, Inner Mongolia autonomous region	2017	NE
RN190	Chifeng city, Inner Mongolia autonomous region	2017	NE
RN191	Chifeng city, Inner Mongolia autonomous region	2017	NE
RN192	Chifeng city, Inner Mongolia autonomous region	2017	NE
RN193	Chifeng city, Inner Mongolia autonomous region	2017	NE
RN194	Chifeng city, Inner Mongolia autonomous region	2017	NE
RN195	Chifeng city, Inner Mongolia autonomous region	2017	NE
RGS5	Jiuquan city, Gansu province	2017	NW
RGS6	Jiuquan city, Gansu province	2017	NW
RGS7	Jiuquan city, Gansu province	2017	NW
RGS8	Jiuquan city, Gansu province	2017	NW

[a]NE, Northeast China (including Heilongjiang, Jilin province, Chifeng city and Hinggan league of Inner Mongolia autonomous region); NC, Northern China (including Shanxi provinces and Ulanqab city and Baotou city of Inner Mongolia autonomous region); NW, Northwest China (including Gansu province and Xinjiang Uygur autonomous region).
