# Supplementary material for: Genetic Structure of Populations of Rhizoctonia solani Anastomosis Group (AG)-2-2IIIB and AG-4HGI Causing Sugar Beet Root Diseases in China
Source: J Fungi (Basel). 2026 Jan 30;12(2):97. doi: 10.3390/jof12020097 (PMC12941418; doi:10.3390/jof12020097)
Supplement: Supplementary file 1 [file jof-12-00097-s001.zip › Table S3.pdf]

Table S3. Repeat motifs and primer sequences of the 14 loci of simple sequence repeats (SSRs) from *Rhizoctonia solani* AG-2-2IIIB.

SSR loci	Repeat motif	Primer sequence (5′-3′)	Annealing temperature (°C)
C6248	(AAAC)6	F: AGGAACCGGGTATGACAGAA<br>R: TTATTTCTGATTCGGCCGTC	57
C14525	(ACC)8	F: CCCCACTCTATCTCTCTGCG<br>R: GCATTTCTTGCGCCTAACTC	57
C8703	(ACGGAC)7	F: TGTTCCTTCTCACGCTTCCT<br>R: GATTCCGTGTCAAACTCGGT	60
C7683	(AGGTGC)5	F: AGCATAGAATATGGCCGTGC<br>R: GTACACCCACCGAGCTGTTT	59
C15210	(CGAGTC)5	F: TCGTTGATCGATGCGAATAC<br>R: GAAACCTCGCTGTGCCTAAC	57
C8837	(CT)20	F: ACACGCTCTCCCATTATTCG<br>R: GCAAAAGTCAGGACGAGGAG	56
C14161	(GA)9	F: AAGCTAATTGGGTTGGGGTC<br>R: AAGTCAACTGAGCTCGCCAT	59
C9144	(GT)6	F: ACAAGGAAAGCGAGAACGAA<br>R: TTGGTCTTTTTCGGGTTGAG	60
C12183	(TA)10	F: AACAAGATGGTGGGCTTGAC<br>R: TTAGCCGCCTGATCGTAGTT	60
C15253	(TCT)7	F: GATGATTGCTCGGTCAGGAT<br>R: AAAACGAGAGTGGAACGTGG	59
C13740	(TG)7	F: TCGCTTCGTACCCACTCTTT<br>R: ATCTACACTGGCGTCTGCCT	57
C9782	(TGCCT)5	F: TCGTCTTGGATTCTCACGAA<br>R: GCATGCTGTAGCGTTGTGTT	57
C4407	(TTCT)5	F: CCATGATGATGGCTTCTCCT<br>R: ACTGTTGTTGTCGGCTCCTT	59
C14499	(TTGAGC)8	F: CTGTTGTTGTTGTGGCTGCT<br>R: CAAAGCATCTACGGCCAAAT	60
