# Supplementary material for: Genetic Structure of Populations of Rhizoctonia solani Anastomosis Group (AG)-2-2IIIB and AG-4HGI Causing Sugar Beet Root Diseases in China
Source: J Fungi (Basel). 2026 Jan 30;12(2):97. doi: 10.3390/jof12020097 (PMC12941418; doi:10.3390/jof12020097)
Supplement: Supplementary file 1 [file jof-12-00097-s001.zip › Table S4.pdf]

Table S4. Repeat motifs and primer sequences of the 20 loci of simple sequence repeats (SSRs) from *Rhizoctonia solani* AG-4HGI.

SSR loci	Repeat motif	Primer sequence (5′-3′)	Annealing temperature (℃)
012785	(AAAT)5	F: GACACACGATGGTGGATGAG<br>R: AACGGGTTTGGAGGAAAAGT	57
004329	(AAT)5	F: TATACACCGCCTTCTGGACC<br>R: GCACCTTGATTTCCCTGTGT	60
015286	(AATTT)5	F: ACTCGGGTTGATGAGCTTTG<br>R: GTGAATTCAGACCGCTTTGG	57
023115	(AG)11	F: GTGCCGAGTACGGTTTTGAT<br>R: GGTTTCAAGGACCACAAGGA	60
057704	(ACG)6	F: AGAACCGGGAGCTAACCATT<br>R: GATGAGTTTCTGGCGAGGAG	60
006128	(AAG)5	F: ATGCCCTTGGGAGTTTTCTT<br>R: AATGACGTTCGAGGTCGTTC	57
007713	(ACC)5	F: ACCTGGTTCGGGCTTAAAAT<br>R: ATGTGTGGATTGTGCCCATA	60
010525	(AGA)6	F: GGTTCAACTGCCCATCCTAA<br>R: TCCTCAGTGTTGATGGTGGA	60
004651	(ATCC)5	F: TTCACCATCATGTTGCACCT<br>R: ATCATTTCCACCGTCTGCTC	60
063922	(CTG)8	F: GTGACGCATGAGAGTAGGCA<br>R: TGCCCAACGTACATTCTCAA	60
016188	(AGCAGG)6	F: GATAGGAGAGGTCGAAGCCC<br>R: TTCCAAATAATCCGCACCTC	59
068450	(CCTGCT)14	F: ACTCCAGCCACCTCTGTGAG<br>R: GATAGGAGAGGTCGAAGCCC	60
012305	(CT)11	F: TGTACAGGGTGATTGCCTCA<br>R: CTGGCGACTAAACGAAGGAC	60
013519	(CA)10	F: CGTCATGCTATACTCGGCAA<br>R: AATCCATCGGTGGTTCACAT	60
005937	(AGT)5	F: ATGTTACCAATGGCCTCGAC<br>R: AGACAAATCACGGACCAAGG	59
060842	(ATC)5	F: TGAATTCAACCAAGCGTTCA<br>R: GGATTCAGGAAAGTATGGCG	59
042482	(ATT)5	F: ATTTTCCAGCGCATTTTACG<br>R: CATCATCCGTGTCCACACTC	57
022653	(CAC)5	F: GGAATAGAGCGCTACGACCA<br>R: TTTGGGATAACGCCAAAGTC	59
058474	(CAG)8	F: GATGATTGATCAGCTTGGGG<br>R: CCTCATCTGGGAAGTGGGTA	60
011009	(CAT)6	F: GGCAGTTTCCATTCGGATAA<br>R: AGCTGTTGCGAGTGTGATTG	59
