# Supplementary material for: Genetic Structure of Populations of Rhizoctonia solani Anastomosis Group (AG)-2-2IIIB and AG-4HGI Causing Sugar Beet Root Diseases in China
Source: J Fungi (Basel). 2026 Jan 30;12(2):97. doi: 10.3390/jof12020097 (PMC12941418; doi:10.3390/jof12020097)
Supplement: Supplementary file 1 [file jof-12-00097-s001.zip › Table S5.pdf]

Table S5. The size of amplicons, number of strains, and selective neutrality test of *Rhizoctonia solani* AG-2-2IIIB using fourteen markers of simple sequence repeats (SSRs).

SSR loci	SSR (bp)	Number of strains	Test for neutrality				
			OF [a]	Mean	SE [b]	L95 [c]	U95 [d]
C6248	219-247	134	0.4324	0.4383	0.0271	0.2267	0.8455
C14525	258-279	134	0.3120	0.3879	0.0201	0.1972	0.7759
C8703	138-180	134	0.2130	0.3952	0.0204	0.2010	0.7417
C7683	247-289	133	0.4173	0.4327	0.0231	0.2326	0.7903
C15210	240-270	134	0.3789	0.4810	0.0284	0.2451	0.8584
C8837	238-266	134	0.1589	0.2427	0.0082	0.1293	0.4767
C14161	225-243	134	0.2837	0.3610	0.0177	0.1871	0.6988
C9144	243-255	134	0.8450	0.5455	0.0315	0.2802	0.9204
C12183	211-233	133	0.2106	0.3060	0.0130	0.1559	0.6067
C15253	252-279	132	0.7910	0.4907	0.0301	0.2486	0.8699
C13740	234-242	132	0.3048	0.4871	0.0279	0.2482	0.8631
C9782	171-236	133	0.3705	0.3047	0.0136	0.1629	0.6259
C4407	229-253	134	0.5623	0.4888	0.0282	0.2517	0.8657
C14499	235-301	133	0.3347	0.3592	0.0178	0.1876	0.7127

[a] OF, Observed frequency of marker.

[b] SE, Standard error of the mean.

[c] L95, Lower 95% confidence limit.

[d] U95, Upper 95% confidence limit.
