# Supplementary material for: Genetic Structure of Populations of Rhizoctonia solani Anastomosis Group (AG)-2-2IIIB and AG-4HGI Causing Sugar Beet Root Diseases in China
Source: J Fungi (Basel). 2026 Jan 30;12(2):97. doi: 10.3390/jof12020097 (PMC12941418; doi:10.3390/jof12020097)
Supplement: Supplementary file 1 [file jof-12-00097-s001.zip › Table S6.pdf]

Table S6. The size of amplicon, number of strains, and selective neutrality test of *Rhizoctonia solani* AG-4HGI using twenty markers of simple sequence repeats (SSRs).

SSR loci	SSR (bp)	Number of strains	Test for neutrality				
			OF [a]	Mean	SE [b]	L95 [c]	U95 [d]
012785	161-177	145	0.3775	0.5456	0.0325	0.2794	0.9067
004329	189-192	145	0.8424	0.8376	0.0290	0.5029	0.9931
015286	229-249	145	0.6975	0.6313	0.0363	0.3274	0.9593
023115	133-151	145	0.2060	0.3306	0.0156	0.1760	0.6712
057704	158-190	145	0.4250	0.4380	0.0258	0.2258	0.8312
006128	165-183	145	0.5092	0.6321	0.0359	0.3309	0.9593
007713	184-232	145	0.7242	0.5469	0.0340	0.2787	0.9130
010525	179-185	145	0.9397	0.7236	0.0362	0.3802	0.9863
004651	196-208	145	0.3498	0.6221	0.0356	0.3217	0.9526
063922	174-213	145	0.3424	0.3290	0.0149	0.1742	0.6405
016188	235-301	144	0.3298	0.3348	0.0161	0.1772	0.6838
068450	211-301	145	0.3297	0.2851	0.0117	0.1539	0.5750
012305	254-276	145	0.4415	0.6163	0.0329	0.3279	0.9526
013519	235-245	145	0.2505	0.4914	0.0278	0.2492	0.8679
005937	260-266	145	0.4865	0.7090	0.0354	0.3864	0.9795
060842	256-265	145	0.8832	0.7228	0.0363	0.3826	0.9863
042482	206-215	145	0.4042	0.6289	0.0359	0.3365	0.9593
022653	297-315	145	0.3754	0.5418	0.0326	0.2811	0.9130
058474	230-266	145	0.2854	0.3600	0.0171	0.1867	0.6966
011009	169-178	145	0.9660	0.7270	0.0361	0.3898	0.9863

[a] OF, Observed frequency of marker.

[b] SE, Standard error of the mean.

[c] L95, Lower 95% confidence limit.

[d] U95, Upper 95% confidence limit.
